# Improving Ultrasonic Power Transfer in Air Through Hybrid S-Parameter Modeling and High-Efficiency Compensation

**DOI:** 10.3390/s25113340

**Published:** 2025-05-26

**Authors:** Liu Liu, Waleed H. Abdulla

**Affiliations:** Department of Electrical, Computer, and Software Engineering, The University of Auckland, Auckland 1010, New Zealand

**Keywords:** Ultrasonic Power Transfer (UPT), S-parameter, Smith chart, hybrid S-parameter model

## Abstract

Ultrasonic Power Transfer (UPT) offers several advantages over electromagnetic-based wireless power transfer (WPT), but its implementation in the air still faces significant challenges. The low transmission efficiency caused by substantial acoustic energy scattering and absorption and limited output power restricts its use in high-power scenarios. Electrical compensation has proven effective in improving circuit-level performance among various optimization methods, yet its application in air UPT remains underexplored due to the lack of an accurate mathematical model. Traditional modeling approaches, such as the Butterworth–Van Dyke (BVD) model, are unsuitable for air-based UPT systems due to weak coupling and high energy loss. To address these limitations, this paper presents a novel hybrid S-parameter model approach by integrating S-parameter theory with two-port network analysis to improve accuracy and reduce complexity. Based on this model, a novel double-side CL compensation scheme was designed, significantly enhancing the UPT system’s performance while simplifying the compensation circuit design using the Smith chart. Experimental results demonstrate that the proposed scheme enhances efficiency to 2.14% and increases output power to 13.5 mW, significantly improving the transmission performance of the UPT system in the air and offering an effective and practical solution.

## 1. Introduction

Wireless power transfer (WPT) enables energy transmission without direct electrical contact by utilizing either electromagnetic or mechanical energy. Electromagnetic WPT includes magnetic field-based methods, such as inductive coupling, which is widely used for charging electric vehicles [1] and powering medical devices [2], as well as far-field techniques like microwave [3] and laser-based transmission [4]. On the other hand, mechanical WPT transmits energy via acoustic waves—particularly ultrasound—through solid, liquid, or gaseous media, making it especially suitable for environments where electromagnetic transmission is restricted, such as sealed enclosures or biological tissues [5].

In most Ultrasonic Power Transfer (UPT) applications, an air gap is inevitable and significantly affects transmission performance. Traditional electromagnetic-based wireless power transfer (WPT) methods, such as IPT, are highly vulnerable to electromagnetic interference (EMI) when operating across air gaps. In contrast, UPT relies on mechanical waves for energy transmission, which inherently reduces susceptibility to EMI. This makes UPT particularly suitable for applications with stringent electromagnetic compatibility requirements, such as medical devices and aerospace systems. Moreover, UPT can deliver power through relatively small transmitters over longer distances, enhancing its practicality in scenarios demanding compact and efficient wireless power solutions, such as intelligent sensor networks and implantable medical devices [6,7,8]. Therefore, a comprehensive study of UPT performance in air is essential for optimizing WPT technologies and expanding their applicability across various fields.

Despite their advantages, UPT systems in the air still faces several significant challenges. First, transmission efficiency remains relatively low due to substantial scattering and absorption of acoustic energy in air, resulting in considerable energy loss. Second, the output power of UPT systems is limited, making it challenging to satisfy high-power requirements and thus constraining their application in specific scenarios. As a result, improving transmission efficiency and increasing output power remain essential challenges for the continued development of UPT technology. Among various optimization approaches, electrical compensation has been widely recognized for its effectiveness in enhancing circuit-level performance. However, its application in airborne UPT systems has received limited attention, primarily due to the absence of an accurate mathematical model. This lack of modeling constrains the development of systematic design methods and optimization strategies, thereby impeding further progress in UPT system performance.

In conventional applications involving solid media, the Butterworth–Van Dyke (BVD) model is widely used to construct the equivalent circuit of the piezoelectric transducer (PZT) [9,10,11]. The coupling coefficient *M* is typically employed to characterize the interaction between the transmission medium and the PZT [12]. Due to the strong coupling effect, this coefficient can be determined analytically in solid media, such as metals. However, the coupling is significantly weaker in non-solid media like air due to substantial acoustic energy loss during wave propagation. As a result, traditional modeling approaches based on the BVD model are unsuitable for accurately representing UPT systems operating in the air.

To address these challenges, this paper presents a novel modeling approach for UPT systems in the air based on S-parameters. By integrating generalized S-parameter theory [13] with two-port network analysis, a hybrid S-parameter model is developed, which applies to arbitrary UPT systems with any type of compensation network. In the proposed hybrid S-parameter model, losses such as air absorption and nonlinear acoustic effects are not explicitly modeled as independent physical mechanisms. Instead, their overall impact is inherently embedded in the measured or simulated S-parameters used for system characterization. Therefore, the model remains valid as long as the S-parameters are obtained under conditions consistent with practical applications.

Building on this modeling framework, a new compensation circuit is designed specifically for airborne UPT systems to simultaneously enhance output power and transmission efficiency. This approach resolves the shortcomings of conventional modeling techniques in the air media and simplifies the compensation circuit design through the use of the Smith chart. Finally, an experimental prototype was developed, and the results verify that the proposed compensation network significantly improves both power transfer and efficiency in airborne UPT systems.

## 2. S-Parameter Modeling

### 2.1. Transmission Characteristics of the System

**Figure 1:**
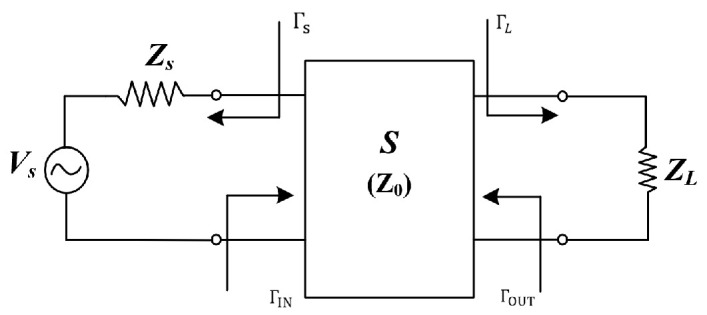
S-parameter model of UPT system [13].

The general equivalent model of the UPT system is illustrated in Figure 1 and comprises the power supply *V_s_*, its internal resistance *Z_s_*, the load *Z_L_*, the PZT, and the transmission medium. The PZT and the transmission medium are characterized by S-parameters.

The reflection coefficient $\Gamma_{IN}$ from the power supply end to the load is shown in Equation (1).

(1) $$\Gamma_{IN}=S_{11}+\frac{S_{12}S_{21}\Gamma_{L}}{1-S_{22}\Gamma_{L}}$$

where $\Gamma_{L}=\frac{Z_{L}-Z_{o}}{Z_{L}+Z_{o}}$. Similarly, the output reflection coefficient $\Gamma_{OUT}$ from the load to the power supply is shown in Equation (2).

(2) $$\Gamma_{OUT}=S_{22}+\frac{S_{12}S_{21}\Gamma_{S}}{1-S_{11}\Gamma_{S}}$$

where $\Gamma_{s}=\frac{Z_{s}-Z_{0}}{Z_{s}+Z_{0}}$, therefore, the input power of the system is

(3) $$P_{IN}=\frac{1}{2}|a_{1}{|}^{2}-\frac{1}{2}|b_{1}{|}^{2}=\frac{1}{2}|a_{1}{|}^{2}(1-|\Gamma_{IN}{|}^{2})$$

Alternatively, the system’s equivalent input impedance, *Z*_in_, can be determined from the reflection coefficient. Combining this with the input voltage can also calculate the system’s output power.

(4) $$P_{L}=\frac{1}{2}|b_{2}{|}^{2}-\frac{1}{2}|a_{2}{|}^{2}=\frac{1}{2}|b_{2}{|}^{2}(1-|\Gamma_{L}{|}^{2})$$

The transmission efficiency of the system can be expressed as

(5) $$\eta =\frac{1}{1-|\Gamma_{IN}{|}^{2}}|S_{21}{|}^{2}\frac{1-|\Gamma_{L}{|}^{2}}{|1-S_{22}\Gamma_{L}{|}^{2}}$$

As can be observed, reducing the reflection coefficients *Γ*_IN_ and *Γ_L_* improves transmission efficiency and increases input power. This can be achieved through a properly designed impedance-compensation circuit. Therefore, an appropriately designed electrical compensation network is essential for optimizing the system’s reflection characteristics and enhancing overall system performance.

### 2.2. Series Compensation

From the perspective of S-parameter theory, an impedance-compensation circuit can match the impedance of the PZT to that of the driving circuit, thereby reducing *S*_11_. A lower *S*_11_ value indicates reduced reflected energy, meaning that more power is absorbed by the transducer and converted into ultrasonic energy or other output forms. In UPT systems, electrical compensation effectively reduces the impedance mismatch between the PZT and the excitation power source, thereby improving output power. Common compensation circuit topologies include series, parallel, *π*, *T*, and *L* configurations. However, any complex compensation network can be constructed by cascading basic series and parallel elements, making these two configurations the theoretical foundation for compensation circuit design.

When the UPT system operates under rated conditions, it can be assumed to function within the linear region. As a result, the UPT system can be equivalently represented by an impedance *Z*_0_ as seen from the excitation power supply. This allows the compensation circuit design to focus exclusively on the impedance relationship between the power supply and *Z*_0_ without considering the internal complexity of the UPT system. This equivalent representation significantly simplifies the design process of the compensation circuit. The S-parameter model of the series compensation circuit is derived below.

Since this is a reciprocal symmetric network, we have

(6) $$\begin{array}{c} S_{11}=S_{22}\\ S_{12}=S_{21} \end{array}$$

where *S*_11_ is the voltage reflection coefficient of the input port when the output port is matched, that is

(7) $$S_{11}=\Gamma_{IN}=\frac{Z_{IN}-Z_{0}}{Z_{IN}+Z_{0}}$$

Among them, $Z_{IN}=Z+Z_{0}$, so we have

(8) $$S_{11}=\frac{Z}{Z+2Z_{0}}$$

(9) $$S_{21}=\frac{V_{2}^{-}}{V_{1}^{+}}{|}_{V_{2}^{+}=0}$$

From Equation (9), we can see that *S*_21_ is the voltage gain when the output ports are matched. If an excitation voltage *E*_1_ is applied to port 1, the output response can be obtained as follows:

(10) $$I=\frac{E_{1}}{Z_{0}+Z_{IN}}$$

(11) $$V_{2}=V_{2}^{-}+V_{2}^{+}$$

Because the load end is matched, that is $V_{2}^{+}=0$,

(12) $$V_{2}^{-}=Z_{0}I_{\cdot }$$

(13) $$V_{1}=V_{1}^{+}+V_{1}^{-}=V_{1}^{+}(1+S_{11})=Z_{IN}I\Rightarrow V_{1}^{+}=\frac{Z_{IN}I}{1+S_{11}}$$

Dividing Equation (12) by Equation (13), we get

(14) $$S_{21}=\frac{V_{2}^{-}}{V_{1}^{+}}=\frac{Z_{0}\left(1+S_{11}\right)}{Z_{IN}}\Rightarrow S_{21}=\frac{2Z_{0}}{Z+2Z_{0}}$$

Therefore, the S matrix of the series matching circuit can be written as

(15) $$S=\left[\begin{array}{cc} \frac{Z}{Z+2Z_{0}} & \frac{2Z_{0}}{Z+2Z_{0}}\\ \frac{2Z_{0}}{Z+2Z_{0}} & \frac{Z}{Z+2Z_{0}} \end{array}\right]$$

**Figure 2:**
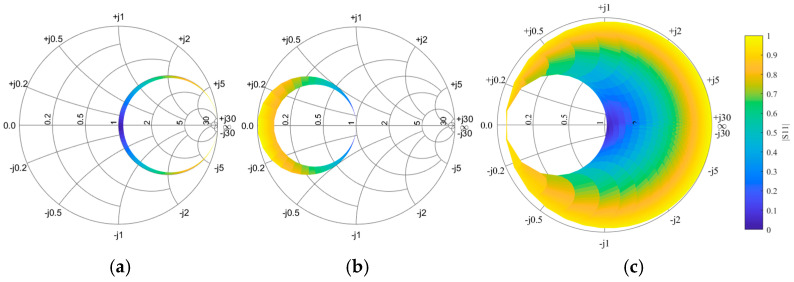
Compensable load impedance range of compensation network (**a**) a series *L*, (**b**) a parallel *L*, (**c**) a CL.

According to Formula (15), we can determine the matching range of the series compensation circuit. Assuming the target matching resistance is 50 ohms, and assuming the series reactance range is *j* (−2000, 2000) ohms, we can get the matching range of the series compensation circuit, as shown in Figure 2 However, this range is relatively small, indicating that the applicability of the series compensation method in practical applications has certain limitations.

### 2.3. Parallel Compensation

The influence of parallel impedance *Y* on S-parameters is reflected in the input reflection coefficient *S*_11_ and the transmission coefficient *S*_21_. The S-parameter model of the parallel compensation circuit is similar to the derivation process of series *L*. Therefore, the S matrix with parallel can be written as shown in Equation (16).

(16) $$S=\left[\begin{array}{cc} \frac{-Z_{0}Y}{2+Z_{0}Y} & \frac{2}{2+Z_{0}Y}\\ \frac{2}{2+Z_{0}Y} & \frac{-Z_{0}Y}{2+Z_{0}Y} \end{array}\right]$$

According to the input impedance of parallel compensation, the matching range of the parallel compensation circuit can be obtained, and the equivalent reactance variation range is *j* (−2000, 2000) ohms, as shown in Figure 2. Compared with the series compensation method, the matching range of parallel compensation is extended and can adapt to a broader range of load changes.

### 2.4. Capacitor–Inductor (CL) Compensation

The design of the compensation structure is not limited to series impedance *Z* or parallel impedance *Y* but can also incorporate cascaded configurations to form a more complex matching circuit. By treating series and parallel circuits as fundamental units and applying the cascading operation rules of S-parameters, the analysis and calculation of the overall network can be significantly simplified.

**Figure 3:**
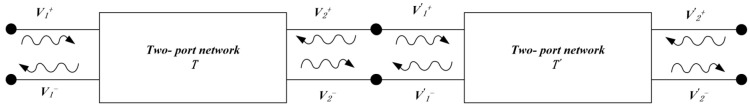
Cascading of two-port networks based on Wave T-parameters.

However, cascading S-parameter matrices is complex because the S-matrix, based on normalized characteristic impedance, does not allow direct matrix multiplication for network characterization. Wave T-parameters are used for intermediate calculations to simplify the analysis, as they enable straightforward cascading through matrix multiplication. The overall T-matrix is then converted back to the S-matrix to determine the S-parameters of the cascaded system. The T-matrix is defined with input port voltage and current as independent variables and output port voltage and current as dependent variables, as shown in Figure 3.

Therefore, when analyzing compensation circuits, the advantages of T- and S parameters can be combined to analyze complex compensation networks efficiently.

The T-matrix of the entire network is obtained by multiplying the T-matrices of the two sub-networks, as shown in Equation (17).

(17) $$T_{total}=TT^{\prime }$$

The conversion relationship between S parameters and Wave T parameters is as follows:

(18) $$\left[\begin{array}{cc} T_{11} & T_{12}\\ T_{21} & T_{22} \end{array}\right]=\left[\begin{array}{cc} \frac{1}{S_{21}} & -\frac{S_{22}}{S_{21}}\\ \frac{S_{11}}{S_{21}} & S_{12}-\frac{S_{11}S_{22}}{S_{21}} \end{array}\right]\;\left[\begin{array}{cc} S_{11} & S_{12}\\ S_{21} & S_{22} \end{array}\right]=\left[\begin{array}{cc} \frac{T_{21}}{T_{11}} & T_{22}-\frac{T_{21}T_{12}}{T_{11}}\\ \frac{1}{T_{11}} & -\frac{T_{12}}{T_{11}} \end{array}\right]$$

Therefore, after deriving the S-parameter models for individual sub-matching circuits, they are converted into their corresponding Wave T-parameter representations. The overall T-matrix is then obtained by multiplying the T-matrices of all interconnected stages. Finally, the total T-matrix is converted back into an S-matrix using Equation (18), yielding the equivalent S-parameters for the entire cascaded compensation circuit. This modeling approach significantly facilitates efficient analysis.

In the design of compensation structures, selecting an appropriate matching network is essential for optimizing power transmission. Traditional series or parallel inductor tuning can compensate for the transducer’s static capacitance but cannot transform impedance. As a result, the input impedance remains fixed, reducing adaptability to varying power supply conditions.

**Figure 4:**
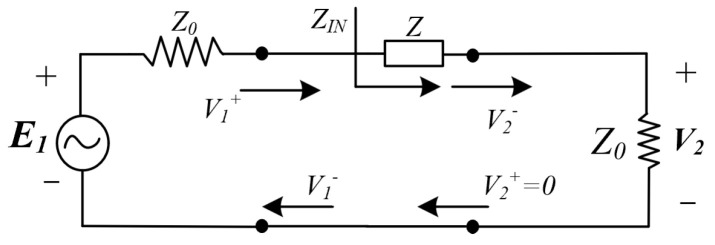
CL compensation network.

The Capacitor–Inductor (CL) compensation circuit, as shown in Figure 4, addresses this limitation by adjusting the equivalent input impedance to achieve better impedance matching. This allows the transducer to extract more power from the source, thereby improving overall energy utilization efficiency. Accordingly, this section presents the design of a CL compensation circuit as an example and derives its corresponding S-parameter model, as given in Equation (19).

(19) $$\left[\begin{array}{cc} S_{11} & S_{12}\\ S_{21} & S_{22} \end{array}\right]=\left[\begin{array}{cc} 1-\frac{2Z_{0}(YZ_{0}+1)}{2Z_{0}+Z\left(YZ_{0}+1\right)+YZ_{0}^{2}} & \frac{2Z_{0}}{Z+2Z_{0}+YZ_{0}(Z+Z_{0})}\\ \frac{2Z_{0}}{Z+2Z_{0}+YZ_{0}(Z+Z_{0})} & \frac{2Z+2Z_{0}}{YZ_{0}^{2}+\left(YZ+2\right)Z_{0}+Z}-1 \end{array}\right]$$

According to Equation (19), assuming the range of series and parallel reactance is *j* [−1000, 1000] Ω, the matching range of the CL compensation circuit can be determined, as illustrated in Figure 2. It is evident that the CL compensation circuit significantly expands the matching range compared to conventional series and parallel compensation methods, with only a small region remaining unmatched. Moreover, the CL compensation circuit combines the high-efficiency power transfer characteristics of series compensation with the load adaptability of parallel compensation, enabling effective impedance matching over a broader load range. This improves system robustness by reducing sensitivity to load variations and frequency shifts, ensuring more stable performance in practical applications. In addition, the CL topology requires a few components, which helps minimize additional losses. Therefore, the CL compensation circuit represents an ideal compensation topology with superior performance characteristics.

### 2.5. Proposed Hybrid S-Parameter Model

**Figure 5:**
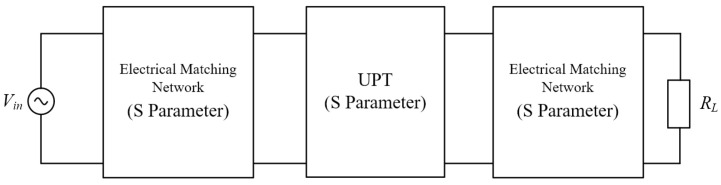
UPT model based on S-parameter.

Equation (5) considers only the system’s S-parameters for estimating efficiency but cannot accurately assess the efficiency of systems incorporating additional matching circuits, thereby limiting the design of such circuits. This section proposes a hybrid mathematical model that integrates circuit S-parameters to overcome this limitation. This model enables precise estimation of both efficiency and power for systems that include arbitrary matching circuits and UPT system S-parameters, as illustrated in Figure 5.

According to Equation (18), the S-parameters are converted into T-parameters. The T-parameters of the total system can be obtained as *T_total_ = T*_1_ *T*_2_ *T*_3_ and converted back to S parameters using Equation (18). To estimate the electrical parameters of the system, such as power efficiency, the S-parameters can be converted into Z-parameters and then into a T-network.

The relationship between S-parameters and Z-parameters is shown in Equation (20).

(20) $$\begin{array}{c} Z_{11}=Z_{0}\cdot \frac{(1+S_{11})(1-S_{22})+S_{12}S_{21}}{(1-S_{11})(1-S_{22})-S_{12}S_{21}}\\ Z_{12}=Z_{0}\cdot \frac{2S_{12}}{(1-S_{11})(1-S_{22})-S_{12}S_{21}}\\ Z_{21}=Z_{0}\cdot \frac{2S_{21}}{(1-S_{11})(1-S_{22})-S_{12}S_{21}}\\ Z_{22}=Z_{0}\cdot \frac{(1-S_{11})(1+S_{22})+S_{12}S_{21}}{(1-S_{11})(1-S_{22})-S_{12}S_{21}} \end{array}$$

**Figure 6:**
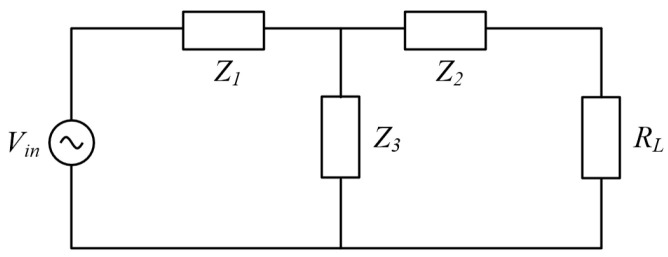
Equivalent circuit based on Z-network.

Assume that the system is connected to a load R_L_ and the input voltage is *V*_in_, as shown in Figure 6.

(21) $$\begin{array}{c} Z_{1}=Z_{11}-Z_{12}\\ Z_{2}=Z_{22}-Z_{21}\\ Z_{3}=Z_{12} \end{array}$$

The primary and secondary currents of the system can be calculated by KVL KCL,

(22) $$\begin{array}{c} I_{1}=\frac{V_{in}\cdot (Z_{2}+Z_{3}+Z_{L})}{Z_{2}Z_{3}+Z_{3}Z_{L}+Z_{1}(Z_{2}+Z_{3}+Z_{L})}\\ I_{2}=\frac{V_{in}\cdot Z_{3}}{Z_{2}Z_{3}+Z_{3}Z_{L}+Z_{1}(Z_{2}+Z_{3}+Z_{L})} \end{array}$$

Therefore, the system input power, output power and efficiency can be expressed as Equation (23).

(23) $$\begin{array}{c} P_{in}=\frac{1}{2}Re[V_{in}I_{1}^{*}]\\ P_{out}=\frac{1}{2}Re[{\left|I_{2}\right|}^{2}R_{L}]\\ \eta =\frac{P_{out}}{P_{in}} \end{array}$$

## 3. Proposed Electrical Compensation Design Method

### 3.1. S-Parameter Measurement

**Figure 7:**
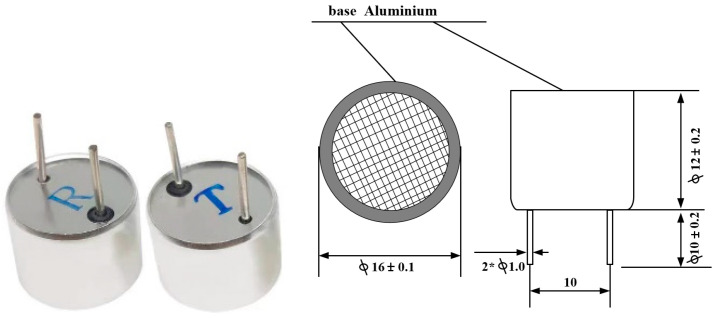
Schematic diagram of TCT40-16T/R-1.2.3.

Accurate modeling of PZT is critical for optimizing the compensation circuit in UPT systems. To extract the electrical characteristics and construct an equivalent model of the UPT, a vector network analyzer (VNA) is used to measure the S-parameters. To improve the general applicability of the analysis, the Air Ultrasonic Ceramic Transducer TCT40-16T/R-1.2.3 is selected as the test device. Its physical appearance and structural diagram are shown in Figure 7.

**Figure 8:**
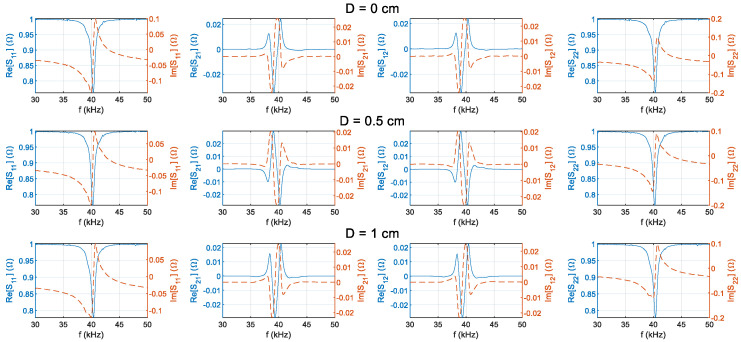

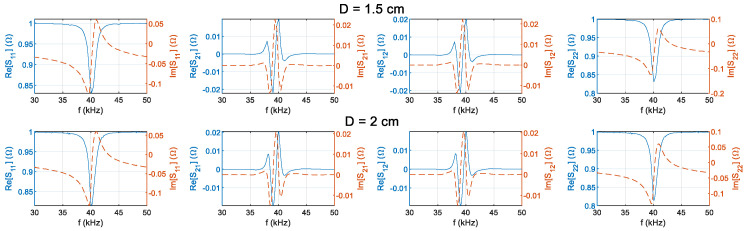
Measured S-parameter frequency responses at different distances.

The S-parameter frequency characteristics were measured from 30 kHz to 50 kHz for the transmitter and receiver at five different distances (0 cm, 0.5 cm, 1 cm, 1.5 cm, and 2 cm). The results are presented in Figure 8. The blue line is the real part of the |*S*|-parameter, and the orange dotted line is the imaginary part of the S-parameter. From left to right: |*S*_11_|, |*S*_12_|, |*S*_21_|, |*S*_22_|. Experimental results indicate that the PZT achieves optimal transmission performance at D = 0.5 cm and f = 39.2 kHz. This is because the modulus of |*S*_21_| is maximum at this distance. Consequently, this distance was chosen as the primary focus for subsequent investigations.

### 3.2. Primary-Side CL Compensation Circuit Design

**Figure 9:**
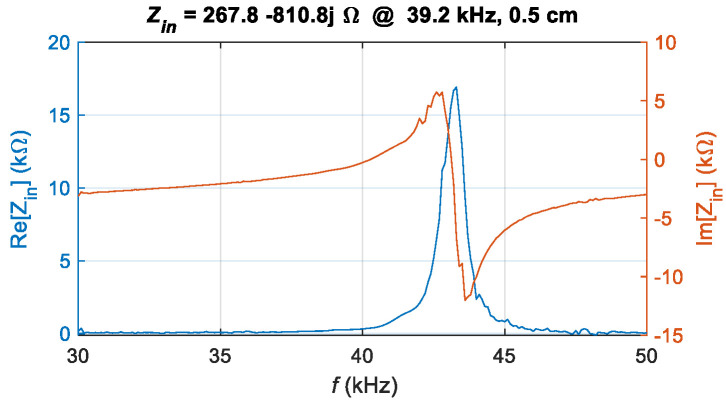
Measured equivalent input impedance *Z*_in_ of the UPT system.

At D = 0.5 cm, the measured |*S*_11_| is −0.3 dB, indicating significant power reflection. Therefore, a matching circuit is required. The design process begins by calculating the system’s equivalent input impedance. The parameter *S*_11_ is directly related to the equivalent input impedance *Z*_in_, and their relationship is given by:

(24) $$Z_{in}=Z_{0}\frac{1+S_{11}}{1-S_{11}}$$

where *Z*_0_ is the characteristic impedance of the system. Accordingly, the frequency characteristics of the equivalent input impedance *Z*_in_ for the UPT system are obtained, as illustrated in Figure 9. The impedance at the peak frequency of *S*_21_, f = 39.2 kHz, is chosen as the matching target, with *Z*_in_ = 267.842 − 813.830 *j*.

**Figure 10:**
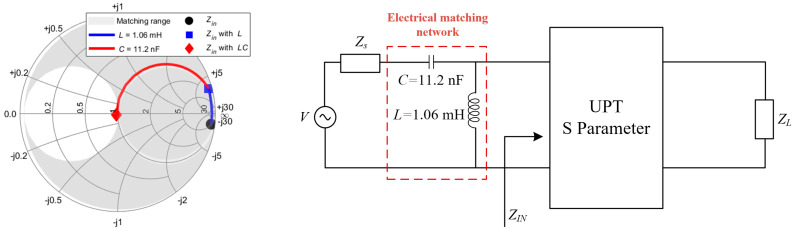
Input impedance *Z*_in_ compensation path and primary-side CL equivalent circuit.

As shown in Figure 10, *Z*_in_ falls within the CL matching range, making the CL topology suitable for matching circuit design. A Smith chart is utilized to perform impedance matching at the input port, ensuring that the equivalent input impedance *Z*_in_ aligns with the target impedance *Z*_0_. It is important to note that *Z*_0_ can be adjusted based on different design objectives. For demonstration purposes, a reference impedance of 50 ohms is used in this case.

**Figure 11:**
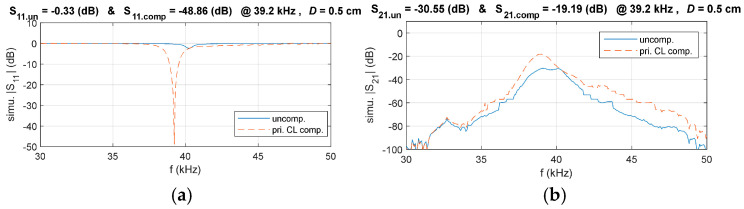
Comparison of simulated *S*_11_ and *S*_21_ in the primary-side CL compensated and uncompensated systems, (**a**) *S*_11_, (**b**) *S*_21_.

Accordingly, the circuit for the primary-side CL compensation circuit is illustrated in Figure 10. To validate the effectiveness of the proposed compensation circuit design method, simulations were conducted to compare the system’s *S*_11_ and *S*_21_ in the compensated and uncompensated matching system. The results are presented in Figure 11.

It can be seen that the reflection coefficient *S*_11_ of the primary matching system is better than that of the uncompensated system. In addition, *S*_21_ is also greatly improved, which proves the effectiveness of the proposed S-modeling method and compensation circuit design method.

### 3.3. Double-Side CL Compensation Circuit Design

**Figure 12:**
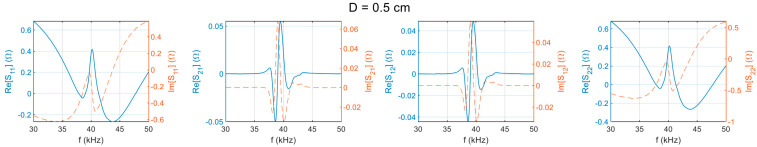
Measured S-parameters of UPT system with primary-side compensation at D = 0.5 cm.

While the primary-side electrical matching network has effectively minimized *Γ*_IN_, further efficiency enhancement requires proper matching of Γ_L_ on the secondary side. As stated in Equation (5), reducing both *Γ*_IN_ and *Γ_L_* contributes to improved system efficiency. The S-parameters of the UPT system with the primary-side CL compensation circuit are shown in Figure 12. Measurement results show that at D = 0.5 cm, the magnitude of |*S*_22_| is −1.1 dB, indicating considerable reflection at the output. To enhance system performance, a secondary-side matching circuit is required.

**Figure 13:**
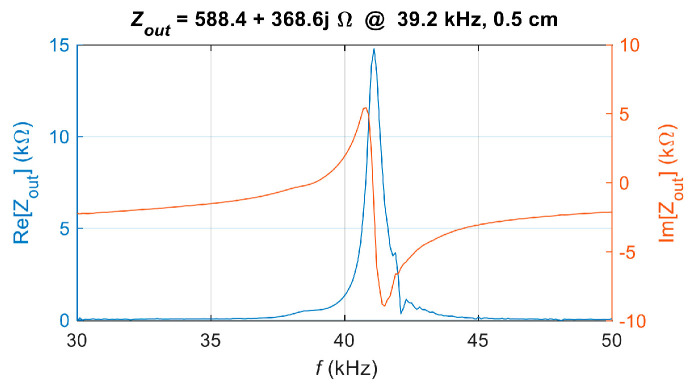
Measured equivalent output impedance *Z*_out_ of the UPT system.

Given that *S*_22_ is directly correlated with the equivalent output impedance *Z*_out_, the system’s equivalent output impedance can be determined from the relationship between impedance and *S*_22_, which is defined as:

(25) $$Z_{out}=Z_{0}\frac{1+S_{22}}{1-S_{22}}$$

where *Z*_0_ is the characteristic impedance of the system. Accordingly, the frequency characteristics of the equivalent output impedance *Z*_out_ for the UPT system can be obtained, as shown in Figure 13. The impedance at the frequency corresponding to the peak *S*_12_, *f* = 39.2 kHz, is selected as the matching target, with *Z*_out_ = 588.43 + 368.552*j*.

**Figure 14:**
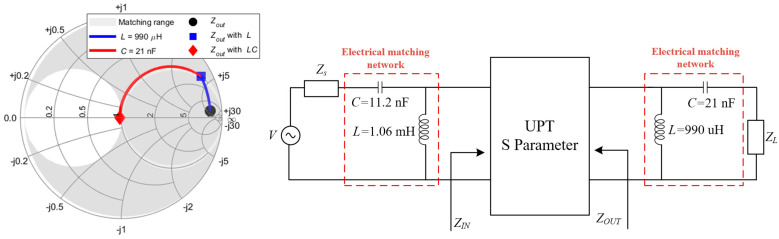
Output impedance *Z*_out_ compensation path and double-side CL equivalent circuit.

As shown in Figure 14, *Z*_out_ falls within the CL matching range, making the CL topology suitable for matching circuit design. A Smith chart is employed to achieve the impedance matching at the output port, ensuring that the equivalent output impedance *Z*_out_ is equal to the target impedance *Z*_0_. It is important to note that *Z*_0_ can be adjusted according to different design objectives. For demonstration purposes, a reference impedance of 50 ohms is used in this case.

**Figure 15:**
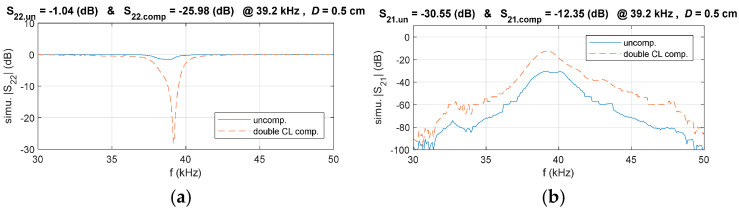
Comparison of simulated *S*_22_ and *S*_21_ in the double-side CL and uncompensated systems, (**a**) *S*_22_, (**b**) *S*_21_.

As a result, the circuit for the double-side CL compensation circuit is derived and illustrated in Figure 14. To validate the effectiveness of the proposed compensation circuit design method, simulations were conducted to compare the system’s *S*_22_ and *S*_21_ in the double-compensated CL and uncompensated CL systems. The results are presented in Figure 15.

To validate the effectiveness of the proposed compensation circuit design method, simulations were conducted to compare the system’s S22 and S21 in both the double-compensated CL and uncompensated configurations. The results are presented in Figure 15. It can be observed that the reflection coefficient S22 of the double-side CL compensation system is significantly reduced compared to the uncompensated system. Additionally, S21 shows a substantial improvement, demonstrating the effectiveness of the proposed S-parameter modeling approach and compensation circuit design method.

### 3.4. Relationship Between Power, Efficiency, Z_0_, and R_L_

To illustrate the proposed compensation circuit design method without loss of generality, the previous matching examples were based on a reference impedance of *Z*_0_ = 50 Ω. However, in practical scenarios, the compensation target should be adjusted according to the load conditions to maximize power transfer and system efficiency. This is because the output power and efficiency depend on the values of *Z*_0_ and the load resistance *R_L_*. The current system design is primarily intended for fixed-load applications, such as powering sensors. To extend its applicability to scenarios with varying load conditions, well-established impedance adaptation techniques can be integrated, including: (1) tunable impedance matching networks [14,15], (2) DC–DC converters on the load side used as impedance transformers [16,17], (3) frequency tracking methods [18,19], and (4) active rectifiers on the load side for adaptive impedance matching [20,21].

**Figure 16:**
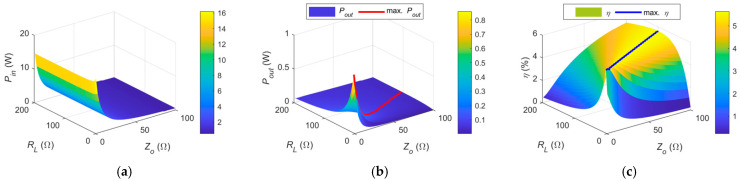
Relationship of *Z*_0_ and *R_L_* with (**a**) input power, (**b**) output power, and (**c**) efficiency in a double-side CL compensated system at D = 0.5 cm.

To clarify this relationship, the influence of Z_0_ and R_L_ on system power transmission and efficiency is further analyzed. Based on the proposed hybrid S-parameter model, the input power (*P*_in_), output power (*P*_out_), and efficiency (*η*) are plotted with a fixed transmission distance of D = 0.5 cm. The values of *Z*_0_ and *R_L_* are varied within the ranges [0, 100] Ω and [0, 200] Ω, respectively. The variation of *Z*_0_ is achieved by adjusting the primary- and secondary-side capacitance values and inductance values in the double-side CL compensation network. For better visualization, the curves of maximum P_out_ and maximum *η* are also included, as shown in Figure 16.

It can be observed that the input power *P*_in_ is primarily affected by *Z*_0_, where a smaller *Z*_0_ results in higher input power. The output power *P*_out_ is influenced by both *Z*_0_ and *Z_L_*, with lower values leading to higher received power. Furthermore, the red curve representing the maximum *P*_out_ indicates that the highest output power is achieved when *Z*_0_ is approximately equal to *R_L_* for a specific *R_L_*.

**Figure 17:**
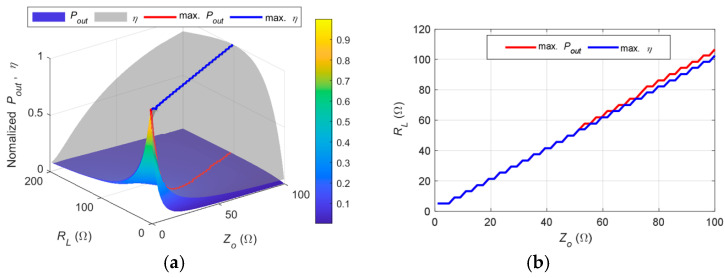
(**a**) Relationship of normalized output power, normalized efficiency, maximum output power, and maximum efficiency with *Z*_0_ and *R_L_*; (**b**) comparison of the corresponding *Z_L_* and *Z*_0_ for maximum output power and efficiency.

Efficiency increases with *Z*_0_, and similarly, maximum efficiency is obtained when *Z*_0_ closely matches *R_L_*. To provide a clearer comparison, the normalized *P*_out_ and *η* and their respective maximum curves are also plotted. In addition, a comparison of the corresponding *Z*_0_ and *R_L_* values at which the maximum *P*_out_ and *η* occur is presented, as shown in Figure 17.

It can be observed that the curves of maximum *P*_out_ and maximum *η* are nearly identical. Therefore, for a given load *R_L_*, the optimal reference impedance *Z*_0_ should be approximately equal to *R_L_* to maximize both the received power and efficiency.

## 4. Results

### 4.1. Primary-Side CL Compensation Circuit

**Figure 18:**
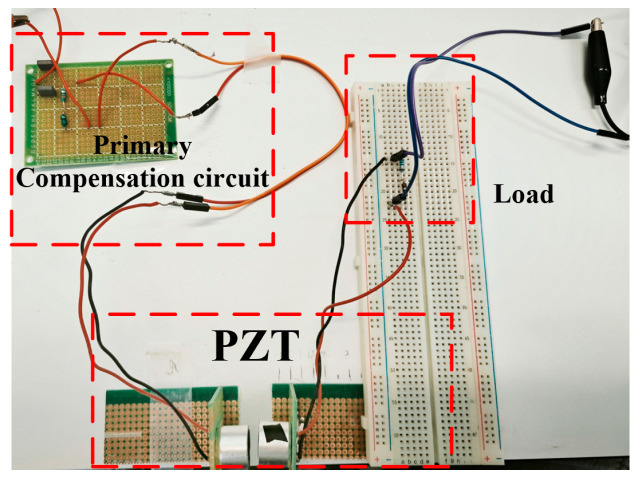
Experimental prototype of primary-side CL compensation circuit.

An experimental prototype was constructed to validate the proposed method, as shown in Figure 18. In this prototype, the values of the selected capacitor and inductor were slightly adjusted from the theoretical calculations. This discrepancy arises because theoretical calculations assume ideal components, whereas practical circuit performance is influenced by equivalent series resistance (ESR), component tolerances, parasitic effects, and measurement equipment inaccuracies. Therefore, experimental tuning and parameter optimization are necessary to achieve optimal matching after obtaining the theoretical reference values. The final matching circuit parameters were determined as *C* = 19.978 nF and *L* = 647.89 uH.

**Figure 19:**
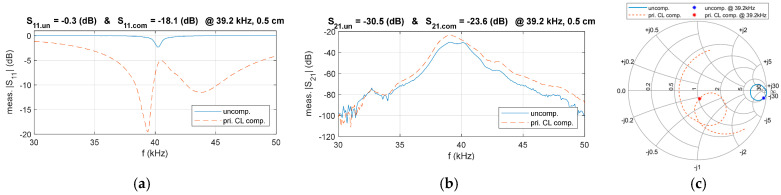
Comparison of measured parameters in the primary-side CL compensated and uncompensated system, (**a**) *S*_11_, (**b**) *S*_21_, (**c**) Smith chart trajectories.

To verify the proposed compensation circuit design, a comparison of the frequency characteristics of *S*_11_ before and after matching was conducted, as illustrated in Figure 19. The results show that, at 39.2 kHz, the magnitude of *S*_11_ decreased from −0.3 dB to −18.1 dB, indicating a significant reduction in input port reflection loss. Meanwhile, the magnitude of *S*_21_ increased from −30.5 dB to −23.6 dB, demonstrating an improvement in transmission efficiency. These findings validate the matching circuit’s effectiveness and the proposed design method.

The experimental results were plotted on the Smith chart to gain further insight into the matching performance, as illustrated in Figure 19c. It can be observed that the designed CL compensation circuit effectively aligns the system’s input impedance with the target impedance, leading to a significant reduction in the magnitude of *S*_11_.

**Figure 20:**
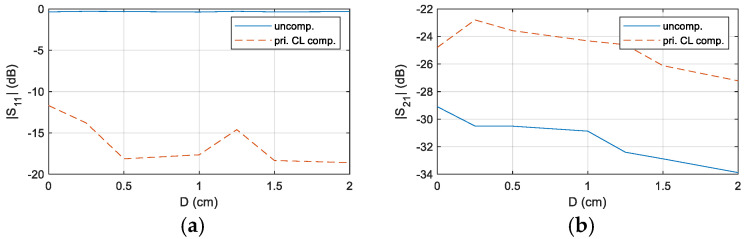
Comparison of *S*_11_ and *S*_21_ in the primary-side CL compensated and uncompensated CL matching system at different distances, (**a**) $\left|S_{11}\right|$, (**b**) $\left|S_{21}\right|$.

The results confirm that the system’s transmission performance is significantly enhanced after impedance matching at a distance of D = 0.5 cm. To further demonstrate the versatility of the designed compensation circuit, the experiment was extended to cover distances ranging from 0 cm to 2 cm. A comparative analysis of *S*_11_ and *S*_21_ before and after compensation at f = 39.2 kHz was conducted, and the corresponding results are presented in Figure 20.

The results indicate that, with the compensation circuit, the magnitude of *S*_11_ is significantly reduced across all measured distances, while *S*_21_ exhibits a notable increase. This confirms the robustness of the designed compensation circuit and the proposed design method, as they effectively mitigate impedance mismatches and enhance system performance under varying distance conditions.

### 4.2. Double-Side CL Compensation Circuit

**Figure 21:**
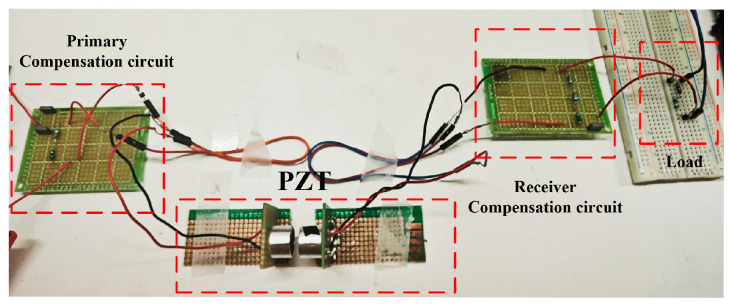
Experimental prototype of double-side CL compensation circuit.

An experimental prototype was developed to verify the proposed method, as illustrated in Figure 21. In this prototype, the values of the selected capacitor and inductor were slightly adjusted compared to their theoretical values. These deviations primarily stem from the fact that theoretical calculations assume ideal components, while practical circuit performance is affected by factors such as ESR, component tolerances, parasitic effects, and measurement equipment inaccuracies. Thus, after obtaining theoretical reference values, experimental tuning and parameter optimization are required to achieve optimal matching. The final compensation circuit parameters were determined as *C* = 20.942 nF and *L* = 841.24 uH.

**Figure 22:**
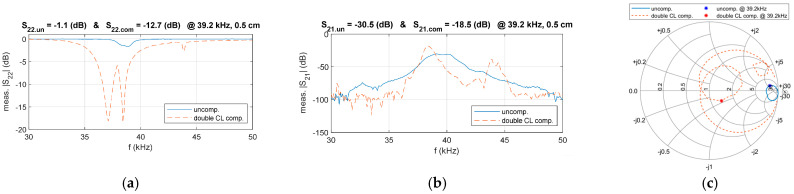
Comparison of measured parameters in the double-side CL compensated and uncompensated system, (**a**) *S*_22_, (**b**) *S*_21_, (**c**) Smith chart trajectories.

To verify the proposed compensation circuit design, a comparison of the frequency characteristics of *S*_22_ and *S*_21_ before and after compensation was conducted, as illustrated in Figure 22. The results indicate that, after compensation, the magnitude of *S*_22_ decreased from −1.1 dB to −12.7 dB at 39.2 kHz, demonstrating the effectiveness of the compensation circuit. Additionally, the magnitude of *S*_21_ significantly improved, increasing from −30.7 dB to −18.7 dB at 39.2 kHz.

The experimental results were plotted on the Smith chart to gain further insight into the compensation performance, as illustrated in Figure 22c. It can be observed that the designed double-side CL compensation circuit effectively aligns the system’s output impedance with the target impedance, leading to a significant reduction in the magnitude of *S*_22_.

**Figure 23:**
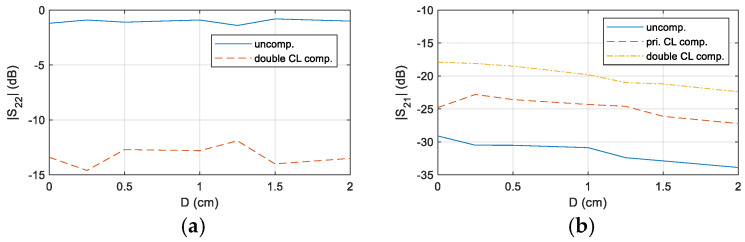
Comparison of *S*_22_ and *S*_21_ in the double-compensated CL and uncompensated CL matching system at different distances, (**a**) $\left|S_{22}\right|$, (**b**) $\left|S_{21}\right|$.

The results confirm that, at D = 0.5 cm, the system’s transmission performance is significantly enhanced after impedance matching. To further demonstrate the versatility of the designed compensation circuit, the experiment was extended to cover distances from 0 cm to 2 cm. A comparative analysis of *S*_22_ and *S*_21_ at f = 39.2 kHz was conducted under three conditions: uncompensation, primary-side CL compensation, and double-side CL compensation. The corresponding results are presented in Figure 23.

The results show that, across different distances, the system with double-side CL compensation achieves the lowest *S*_22_ and the highest *S*_21_, highlighting the critical role of proper compensation design in optimizing transmission performance.

### 4.3. Time-Domain Waveform Comparison

**Figure 24:**
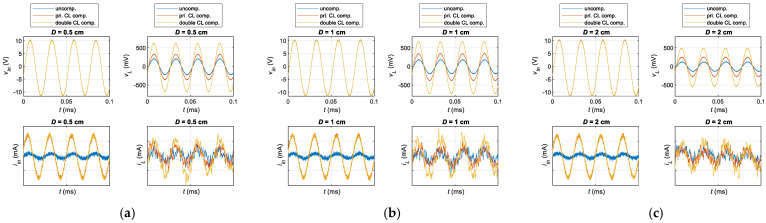
Comparison of the measured waveforms of input voltage, output voltage, input current, and output current in the time domain for the uncompensated, primary-side CL, and double-side CL compensated systems at (**a**) D = 0.5 cm, (**b**) D = 1 cm, and (**c**) D = 2 cm.

Time-domain voltage and current waveforms were measured and analyzed at different distances (D = 0.5 cm, 1 cm, and 2 cm) to validate the performance enhancement further. A comparative study was conducted under three configurations: uncompensation, primary-side CL compensation, and dual-side CL compensation, as presented in Figure 24. For a fair comparison, the sinusoidal input voltage was maintained at *V_pp_* = 21 V, and the load resistance was set to 50 ohms.

The results reveal that, in all cases, the system with double-side CL compensation achieves the highest output power. The input current of both the primary-side and double-side CL compensation systems significantly increases compared to the uncompensated system, rising from 15.7 mA to nearly 150.1 mA, indicating a substantial improvement in input power. Additionally, the load voltage in the double-side CL compensation system shows the most notable enhancement, increasing from 124.4 mV to 483.23 mV. In contrast, the primary-side CL compensation only raises it to 250.9 mV. A similar trend is observed in the load current, confirming a significant increase in load power with the double-side CL compensation. Moreover, as the transmission distance increases, the improvements in both input and output power remain evident, further validating the effectiveness of the proposed method. It is worth noting that, although the experimental results demonstrate the advantages of the double-side CL compensation circuit in enhancing system efficiency and output power, system performance can be further optimized through iterative, simultaneous tuning of the compensation parameters on both the primary and secondary sides.

### 4.4. Transmission Characteristics Comparison

**Figure 25:**
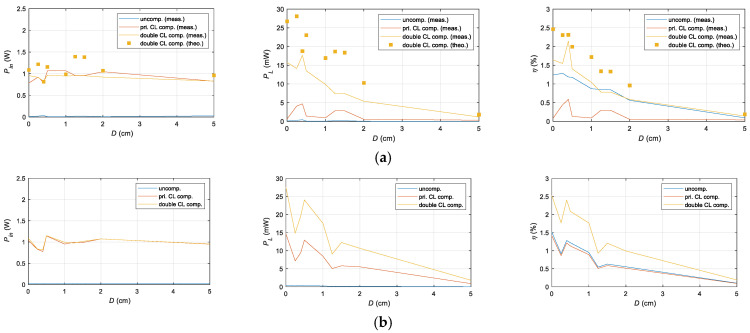
Comparison of input power, output power, and efficiency for the uncompensated, primary-side CL, and double-side CL compensated systems at different distances. (**a**) Measured versus theoretical and (**b**) Simulated.

To further validate the performance enhancement of the UPT system achieved through electrical compensation, the input power, output power, and efficiency of the uncompensated, primary-side CL compensated, and double-side CL compensated systems were compared at seven different distances. The results are presented in Figure 25.

Primary-side compensation also demonstrated a noticeable increase in input and output power compared to the uncompensated system. At D = 0.5 cm, the input power increased from 7 mW in the uncompensated case to 963 mW with primary-side CL compensation and further to 1.1 W with double-side CL compensation. Similarly, the output power improved from 0.1 µW in the uncompensated case to 1.41 µW with primary-side CL compensation and ultimately to 13.5 mW with double-side CL compensation. Efficiency was also significantly enhanced, increasing from 0.13% with primary-side CL compensation to 1.19% in the uncompensated case and reaching 2.14% with double-side CL compensation at D = 0.4 cm.

The results indicate that the double-side CL compensation circuit achieves the highest input and output power across all transmission distances, demonstrating the effectiveness and superiority of the proposed compensation circuit structure and design method. Furthermore, by comparing the MAE and RMSE between the experimental and theoretical results for the Double-CL topology, it was found that the MAE and RMSE of the output power are 0.0078 and 0.0090, respectively, while the MAE and RMSE of the transmission efficiency are 0.0051 and 0.0056, respectively. These results indicate a high level of consistency between the theoretical model and experimental performance, validating the effectiveness and accuracy of the proposed analysis method. Furthermore, they also demonstrate the stability and predictability of the Double-CL topology in practical applications, providing a reliable foundation for future optimization and engineering implementation.

**Figure 26:**
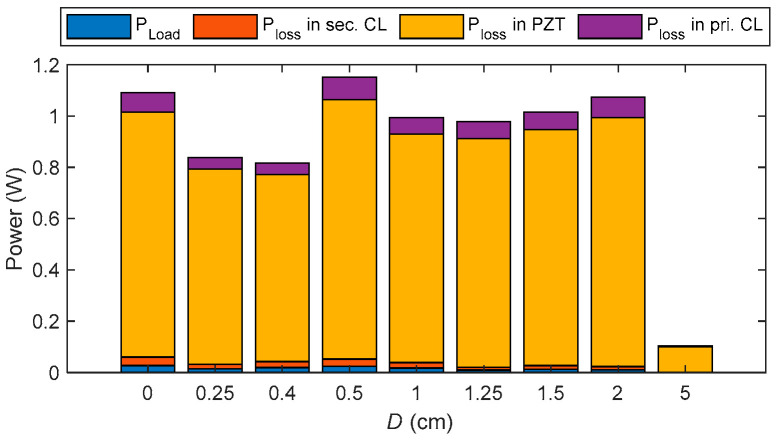
Power Loss Distribution in the UPT System.

In addition, to further demonstrate the superiority of the proposed compensation circuit, the system losses are analyzed. The system’s power distribution was analyzed using a simulation model that has been experimentally validated, as shown in Figure 26. The blue section represents impedance-related losses, the orange indicates losses in the secondary-side CL compensation circuit, the yellow corresponds to the total acoustic transmission loss through the primary PZT, the air medium, and the purple represents losses in the primary-side CL compensation circuit. The figure shows that most energy loss occurs along the acoustic transmission path. The proposed Double-CL compensation circuit significantly enhances power transfer and efficiency while contributing only minimal additional loss, demonstrating its superiority and necessity.

### 4.5. Comparison with Existing Work

This paper compares the results with existing research to verify the superiority of the proposed modeling method and compensation circuit design (see Table 1). It can be observed that current studies on UPT systems in air predominantly focus on acoustic impedance compensation (such as speaker design) and array optimization (enhancing system performance by increasing the number of PZTs). However, research on improving the performance of UPT systems in air through electrical impedance matching remains relatively limited. This gap exists primarily because current research lacks an accurate mathematical model to guide the optimization design of compensation parameters. In this paper, we propose a precise modeling method based on S-parameters, which offers a systematic approach for the electrical compensation of UPT systems in the air, effectively addressing this research gap.

Although the transmission distance in this study is relatively short, this limitation is primarily due to two factors: the PZT’s size and the inductor’s relatively high resistance in the secondary compensation circuit. It is essential to highlight that the compensation method proposed in this paper applies to any PZT, making it highly versatile and suitable for various application scenarios.

## 5. Conclusions

The output power and transmission efficiency of UPT systems in the air have traditionally been very low, limiting their practical applications. A primary cause of this poor performance is the significant impedance mismatch among the PZT, power source, and load. Compensation circuits are commonly introduced between components with mismatched impedances to mitigate this issue. The design of such circuits largely depends on developing an accurate mathematical model of the UPT system. In solid media applications, the BVD model is typically used to model the transmitter and receiver PZTs. The coupling coefficient *M* characterizes the interaction between the transmission medium and the PZT, and it is relatively straightforward to determine in solid materials. However, in the air or other non-solid media, severe acoustic propagation losses significantly weaken the wave coupling effect, making *M* nearly negligible. As a result, it becomes challenging to identify the coupling coefficient in air-based UPT systems, rendering conventional equivalent circuit methods unsuitable for accurate modeling.

To address these limitations, this paper proposes an equivalent model for UPT systems in the air based on S-parameters. This modeling approach avoids the drawbacks of traditional methods and offers benefits such as high accuracy and simplicity. Furthermore, the relationships among S-parameters, system power, and transmission efficiency are derived, leading to a unified model applicable to any compensation network structure. Based on this model, an electrical compensation circuit for UPT systems in the air is developed to improve output power and transmission efficiency. The proposed method simplifies the compensation design using tools like the Smith chart. Building on this modeling and compensation strategy, a double-side CL compensation circuit is proposed to optimize UPT system performance. Firstly, a primary-side CL compensation circuit is implemented to match the equivalent input impedance *Z*_in_ to the target impedance *Z*_0_. An experimental prototype is built to verify the design. After compensation, |*S*_11_| improves from −0.3 dB to −18.1 dB, while |*S*_21_| improves from −30.5 dB to −23.6 dB. These results confirm that primary-side compensation effectively enhances output power.

A secondary-side compensation circuit is introduced to improve performance further, resulting in a double-side CL compensation design. Experimental validation shows that the magnitude of |*S*_22_| decreases from −1.1 dB to −12.7 dB, and |*S*_21_| further improves from −23.6 dB to −18.5 dB. These improvements demonstrate that the proposed compensation approach significantly enhances overall system efficiency. Additionally, time-domain waveform analysis is conducted under three distances. After applying double-side CL compensation, efficiency increases to 2.14%, and output power rises to 13.5 mW. These findings validate the proposed method’s effectiveness in enhancing efficiency and output performance. The impact of transmission distance is also evaluated. Results indicate that the compensated UPT system consistently outperforms the uncompensated system, regardless of whether it operates at the optimal distance. Key performance metrics—efficiency and output power—are improved, confirming that the proposed compensation strategy enhances system robustness against distance variations and increases its practical applicability.

In summary, the proposed double-side CL compensation method significantly improves the output power, transmission efficiency, and environmental adaptability of UPT systems in the air. These contributions offer valuable insights for UPT system optimization and lay the groundwork for future engineering applications.

## Figures and Tables

**Table 1:** Comparison with existing works.

	Medium	Size of PZT	Optimization Method	Improve Power or Efficiency?	*f* (kHz)	*V*in (V)	*P_load_* (mW)	*η*
This work	Air	D = 16 mm	Double-side electrical matching	Both	39.2	10	13.5	2.14% @ 0.5 cm 0.6% @ 2 cm
2003 [22]	Air	D = 36 mm Horn = 65 mm	Acoustic matching horn	Power	28	10	0.05	-
2016 [23]	Air	-	Push–Pull Power Converter	Power	40	7.85	1.071	- 2 cm
2017 [24]	Air	D = 56.4 mm	-	-	50-	-	5 × 10^−3^	- 1.05 m
2018 [25]	Air	7-array D = 45 mm	Array	Efficiency	40	-	-	1.6% @ 5 cm
2020 [26]	Air	-	-	-	50	-	40.5 × 10^−3^	0.32% @ 7 cm
2023 [27]	Magnetic	-	-	-	0.104	289	42.73 × 10^−3^	51.58% @ – cm

## Data Availability

The original contributions presented in this study are included in the article. Further inquiries can be directed to the corresponding author(s).
